# Factors Underlying Food Choice Motives in a Brazilian Sample: The Association with Socioeconomic Factors and Risk Perceptions about Chronic Diseases

**DOI:** 10.3390/foods9081114

**Published:** 2020-08-13

**Authors:** Camila de Mello Marsola, Luís Miguel Cunha, Joana Pereira de Carvalho-Ferreira, Diogo Thimoteo da Cunha

**Affiliations:** 1 Laboratório Multidisciplinar em Alimentos e Saúde, Faculdade de Ciências Aplicadas, Universidade Estadual de Campinas—UNICAMP, R. Pedro Zaccaria, 1300, Limeira SP 13484-350, Brazil; camila.marsola6@gmail.com (C.d.M.M.); joana.ferreira@fca.unicamp.br (J.P.d.C.-F.); 2 GreenUPorto, DGAOT, Faculty of Sciences, University of Porto, Campus Agrário de Vairão, R. da Agrária, 747, 4485–646 Vila do Conde, Portugal; lmcunha@fc.up.pt

**Keywords:** food choice, food consumption, Brazil, exploratory factor analysis, FCQ

## Abstract

This study aimed to evaluate the food choice motives in a sample in Brazil and to identify how socioeconomic characteristics and risk perceptions about chronic diseases and weight gain affect these motives. The Food Choice Questionnaire (FCQ) is an instrument to analyze the motivations for food choice. The FCQ was completed by 525 adult individuals in Brazil. The participants were asked about their perceived risk of gaining weight, developing diabetes, and hypertension. Confirmatory factor analysis led to the rejection of the original FCQ structure, and, after exploratory factor analysis, 30 items of the FCQ were maintained in eight factors: Nutritional Composition, Preparation Convenience, Purchase Convenience, Mood, Sensory Appeal, Health, Price, and Familiarity. Sensory Appeal and Familiarity were, respectively, the most and the least important factors involved in food choices in this sample. A high education level, high income, age, and female sex positively affected FCQ factors (except for the Price factor). On the basis of FCQ scores, we defined five clusters: Health Driven, Practicality Concerned, Shape Concerned, Food Concerned, and Cooking Enthusiasts. In general, individuals were optimistic regarding the risk of developing diabetes, hypertension, and gaining weight, especially those from the Shape Concerned cluster. The differences in food choice motives presented here reinforce the existence of different niches of food consumption. Different types of products can attract specific target groups at the time of choice.

## 1. Introduction

Making choices is a complex mental process. The choice itself is preceded by the judgment of different options available [1]. According to Sobal et al. [2], “food choice involves the selection and consumption of foods and beverages, considering what, how, when, where, and with whom people eat, as well as other aspects of their food and eating behaviors. Food choices play an important role in the symbolic, economic, and social aspects of life by expressing preferences, identities, and cultural meanings”. In this sense, studying food choices is relevant, as it allows for the identification of consumer demand for supplies, consumption of nutrients, and health issues [2], and also because food patterns change over time.

It is known that the nutritional profile of the Brazilian population has transformed in recent decades. Since the 1970s, the prevalence of undernutrition is decreasing while overweight is increasing [3]. Through a survey called VIGITEL (Chronic Disease by Telephone Survey) that was carried out in all capitals of the 26 Brazilian states and the Federal District, researchers monitored the frequency and distribution of risk and protective factors of noncommunicable chronic diseases. An increase in the prevalence of overweight and obesity in all Brazilian capitals was observed through comparing the results of both the 2006 and 2018 surveys [4,5].

Unhealthy food choices are among the leading risk factors for the development of obesity [6,7,8]. As an example, the recent change in the dietary pattern of the Brazilian population is characterized, among other things, by a reduction in the consumption of fresh foods (e.g., fruits, vegetables, beans, and rice) and an increase in the consumption of ultra-processed and ready-to-eat (RTE) foods [4,5,9]. Data from the 2008–2009 Household Budget Survey (*Pesquisa de Orçamentos Familiares*, in Portuguese) [10]—a study carried out in Brazil every 10 years that aims to model the consumption profile of the population—indicated an important reduction in consumption of traditional Brazilian foods such as rice, beans, sweet potatoes, cassava, and fish. At the same time, an increase in RTE food consumption, such as bread, stuffed cookies, frozen food, and soft drinks, was observed in the urban population [10].

Several different aspects can influence consumers’ food choices and the transition in the eating patterns of a population over the years [11,12]. However, it is common for these factors to be studied in isolation, devoting little emphasis on the fact that decisions are interconnected, contextualized, and interspersed with different aspects of the consumer’s life [13]. Therefore, it seems imperative to study the influences, combinations, and sets of factors involved in food choice to understand the connections in this process [13]. Some of those factors are consumer-related as the desire for social approval (i.e., leading the individual to follow social norms at the moment of choice), food consumption in the presence of other people [14], income, nutritional status, appetite, mood, pleasure, desires, and cultural factors [1,13,15]. There are also aspects related to food that affect food choices, such as its intrinsic aspects (e.g., appearance, taste, ingredients, aroma) and extrinsic aspects (e.g., packaging, brand, price, origin, souvenirs) [16,17].

An issue not yet studied is how the perceived risk and control of diseases such as diabetes and hypertension can affect—or be affected by—food choice motives. It is also common that people are concerned about gaining weight, which can affect their practices and choices. Risk perception has also been shown to affect the individual’s behavior directly [15]. Therefore, it is likely that a high perceived risk of food-related diseases would influence individuals to make more prudent and healthier food choices, following the Health Belief Model theory [18]. At the same time, when a hazard is perceived as controllable, individuals tend to be more optimistic and exert riskier behaviors [19].

In order to analyze the motivations for food choices, Steptoe, Pollard, and Wardle created a multidimensional instrument called the Food Choice Questionnaire (FCQ) in 1995. The 36-item FCQ aimed to measure the importance of different motives for daily food choices, which are related to health, price, and convenience, among others [20]. As the FCQ is a multidimensional instrument with a unique rating scale, it is possible to identify, within a population, the factors considered most important for food choice [20].

The FCQ is widely used in several countries to assess the food choice motives within different populations, with cultural adaptations and translations made accordingly [21]. It has already been observed that samples from different countries present different motives for food choice [21]. For example, in a study that compared FCQ answers between four countries, Belgium, Hungary, Romania, and the Philippines, it showed that in the first three, the most important factor for food choice was the sensory appeal. At the same time, for Filipinos, it was the health factor [22]. The hierarchy of factors attributed by the study population appears to depend mainly on the culture, educational level, and circumstances of the countries in which the studies are developed [23]. For example, country-of-origin of foods may affect food choices of Europeans [24], but less likely to affect Brazilians, since they produce most of the food they consume [25]. Moreover, although in some populations, convenience is an important factor in food choice [20], in countries with high consumption of home-made foods and with limited access to the food industry, convenience does not seem to be an important factor [23].

The FCQ was translated into Portuguese and adapted for the Brazilian culture by Heitor et al. [26]. This same research group conducted a study with a Brazilian sample aiming to analyze the dimensional structure and reliability of the FCQ [27]. However, no study was conducted in Brazil to explore other factors underlying food choices, such as risk perceptions, for example.

Considering the above, it is possible to realize the importance of a more in-depth study of the motivations linked to daily food choice considering the complexity of this process in a Brazilian sample. Therefore, the present study aimed to evaluate the food choice motives in a sample in Brazil, to identify whether and how socioeconomic issues affect food choice motives, as well as how food choice motives associate with risk perceptions about chronic diseases and weight gain.

## 2. Materials and Methods

### 2.1. Sample

In this cross-sectional study, individuals over 18 years old from the region of Limeira (São Paulo State, Brazil) were included.

First, a pilot study was carried out with 50 people to estimate the necessary sample. Considering the results, the sample was calculated considering beta error = 1%; alpha error = 5%; effect size = 0.30, requiring 204 participants [28]. As the study involved factor and cluster analysis, as well as regression models, a more robust and conservative sample of 525 individuals was chosen. This sample would allow the use of up to 17 predictors variables in the regression model, following the recommendations of Pedhazur and Schmelkin (1991) [29].

Recruitment took place in different locations to cover different socioeconomic contexts, including a university, public squares, health posts, and condominiums. The participants were recruited face-to-face, using a convenient, intentional, and reasoned sampling with predetermined quotas for age and gender [30]. The sample was collected in order to be equivalent to population characteristics, such as sex (52% female), age (10.5% elderly), and income (50% with income below four times the minimum wage). The education level was categorized as low (incomplete elementary school to incomplete high school) and high (complete high school to complete higher education). The family income was categorized as low (one to four times the minimum wage per month) and high (more than five times the minimum wage per month).

Three trained researchers administered the questionnaires face-to-face. All the participants signed an informed consent form before participating in the study. The Ethics Committee of the State University of Campinas approved the study (CAAE number: 91222418.5.0000.5404).

### 2.2. Evaluation of Food Choices

First, the individuals answered questions about their socioeconomic profiles, such as family income, education level, marital status, and the number of children, in order to characterize the sample.

The FCQ was developed by Steptoe, Pollard, and Wardle [20], and adapted for Brazilian culture by Heitor et al. [26]. The questionnaire has 36 items and is organized initially into nine factors: health, mood, convenience, sensory appeal, natural content, price, weight control, familiarity, and ethical concern. Answers were given using a seven-point scale, with the description in all scale points, ranging from (1) strongly disagree to (7) strongly agree. This scale adaptation was already widely used [23].

### 2.3. Evaluation of Risk and Control Perceptions

The participants were asked about their risk of developing diabetes, hypertension, and gaining weight, as listed below:

R1—What is the chance of you developing diabetes, maintaining your current lifestyle?/Qual a chance de você desenvolver diabetes, mantendo seu estilo de vida atual?
R2—What is the chance of you developing hypertension, maintaining your current lifestyle?/Qual a chance de você desenvolver hipertensão, mantendo seu estilo de vida atual?
R3—What is the chance that you will gain 3 to 10 kg in the next 12 months, maintaining your current lifestyle?/Qual a chance de você engordar 3 a 10 kg nos próximos 12 meses, mantendo seus hábitos atuais?

The risk perception questions were made through considering the recommendation of Helweg-Larsen and Shepperd [31]. The risk perception assessment was measured through a structured psychometric scale with seven options of intensity descriptors from −3 (extremely low) to +3 (extremely high) [32]. All scale points had a specific descriptor.

To verify the perceived control over health and avoiding chronic diseases such as diabetes and hypertension, we used the following two statements:

C1—“I believe I have control over my health”/Eu acredito que tenho controle sobre a minha saúde.
C2—“There is not much I can do to prevent the development of diseases such as diabetes and hypertension”/Não existe muito o que eu possa fazer para evitar o desenvolvimento de doenças como diabetes e hipertensão.

The control statements were made following the same referential as risk perception [31]. Answers were given using the same aforementioned seven-point scale.

### 2.4. Data Analysis

The theoretical distributions of the variables were analyzed using means, deviations, and the histogram of distribution. The Kolmogorov–Smirnov’s test (with Lillefors correction) was used to check normality of data.

The FCQ structure was evaluated by confirmatory factor analysis (CFA) and later by exploratory factor analysis (EFA).

CFA, using a covariance-based structural equation modeling (SEM), was performed to verify the adequacy of the data for the original FCQ structure. The validity of the FCQ factors was assessed using Cronbach’s alpha, with values above 0.60 being considered adequate. The model adjustment was analyzed according to Hu and Bentler [33] using the comparative fit index (CFI) >0.90, standardized root mean squared residual (SRMR) <0.10, root mean square error of approximation (RMSEA) <0.06, and PClose (*p*-value for testing the null hypothesis that RMSEA is no higher than 0.05).

Since an adequate fit of the original FCQ structure was not observed, we performed EFA. In the EFA, valid items from the FCQ were extracted, considering only those with factorial loads above 0.40. EFA was performed with varimax rotation. A Kaiser–Meyer–Olkin (KMO) value greater than 0.70 was used to verify the adequacy. The new model was again submitted to CFA to assess the fit.

The FCQ factors were compared using repeated-measures analysis of variance (ANOVA) with the Bonferroni post hoc test.

Generalized linear models (GLM) were used to determine which variables were associated with food choice factors (normalized variables). First, multiple linear models were developed, however, these models did not present adequate fit. For this reason, linear mixed effect models were chosen. The independent variables in each model were those variables that presented a Pearson correlation coefficient greater than 0.30, and differences in Student’s *t*-test. Homoscedasticity and model fit were evaluated by residual analysis and the chi-squared test.

K-means cluster analysis was used to identify patterns in food choices. The normalized scores for each FCQ factor were included in the analysis. The cluster’s quality was observed through comparing the averages of the scores of each cluster through one-way ANOVA. The comparison of risk perceptions between clusters was made using one-way ANOVA with Bonferroni’s post hoc test. Levene’s test assessed the equality of variance. The clusters’ names were given on the basis of salient factors of FCQ.

The CFA was conducted using the Analysis of Moment Structures—AMOS v.26 software. All the other tests were conducted using the Statistical Package for Social Sciences—SPSS 20.0.1.

## 3. Results

A total of 525 individuals, with a mean age of 34.5 years old (standard deviation (SD) of 16.4), were enrolled in this study. The majority are women (61%) and single (56.8%). The socio-demographic characterization of the sample can be seen in Table 1.

### 3.1. Food Choice Questionnaire’s Structure

The final model, with a better fit, was different from the original FCQ structure. A model with the following indicators was found: CMIN/DF = 0.882; SRMR = 0.067; RMSEA = 0.065; PClose = 0.01; CFI = 0.88. After EFA, 5 of the 36 items originally present were removed. A total of 31 items were kept in the confirmatory factor analysis (CFA), and these comprised the 8 factors (Table 2). According to the rearrangement of the FCQ structure from CFA, some factors were renamed. Nutritional Composition was a new factor formed with items present in the former questionnaire in the Weight Control and Natural Content factors. The items that originally formed the Convenience factor were separated into two new factors named Convenience of Preparation and Convenience of Purchase. The Ethical Concern factor did not appear to be related to the other items evaluated in the instrument and was excluded for a better model fit.

Among the eight factors of the FCQ, Sensory Appeal presented the highest importance score attributed by the sample (Table 1). The factors of Price, Convenience of Purchase, and Health were the second most important, with no differences between them. Thirdly were the factors of Convenience of Preparation, Mood, and Nutritional Composition. Finally, the Familiarity factor was the least important one.

It is possible to observe an adequate discriminant validity, with *r* > 0.30 in most correlations (Table 3). A moderate positive correlation was found between Health and Nutritional Composition.

### 3.2. Food Choice Motives

The mean scores attributed by women, in all factors of the FCQ, were significantly higher (*p* < 0.001) than the scores attributed by men.

Five well-defined clusters were created, namely, Health Driven, Practicality Concerned, Shape Concerned, Food Concerned, and Cooking Enthusiasts (Table 4). The cluster names refer to the FCQ factors with the highest and lowest averages within each cluster. For the cluster named Health Driven, the most salient factors were Health and Convenience of Preparation, and the least important were Familiarity and Mood, indicating the appreciation of healthy food that is easily prepared, but that keeps satisfactory sensory characteristics. In terms of Practicality Concerned, individuals were identified as people who value practicality above other motives when it comes to eating, i.e., prefer food that is easy to buy, easily prepared, and with high sensory appeal. Individuals in this cluster showed lower concern for aspects such as Nutritional Composition and Health. In Shape Concerned, individuals showed concern for food with satisfying sensory characteristics, fair price, and proper nutritional composition. Antagonistically, people in this cluster showed less concern for Health, indicating that the concern with Nutritional Composition is probably related to issues of diet and body weight. In Food Concerned, all factors showed high averages. In this cluster, higher importance was attributed to Nutritional Composition, Preparation Convenience, and Price. Finally, in Cooking Enthusiasts, the individuals showed importance for foods with excellent sensory characteristics that are good for health and that are easy to buy. The counterpoint was the low importance of the practicality of the preparation, suggesting that people in this cluster probably take pleasure in preparing their meals.

### 3.3. Risk Perceptions and the Relationship with Food Choice Motives

**Figure 1:**
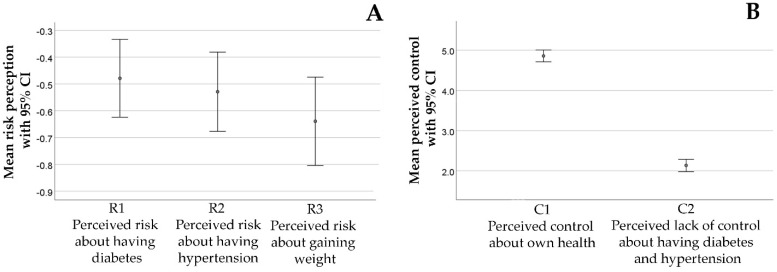
(**A**) Mean risk perception (scaled −3 to 3) about diabetes, hypertension, and gaining weight, with 95% confidence intervals (CI); (**B**) mean perceived control (scaled 1 to 7) about health and having diabetes and hypertension, with 95% confidence intervals (CI).

In general, individuals show an optimistic risk perception of having diabetes, hypertension, and weight gain (Figure 1A), i.e., they perceived, in general, a low personal risk for the indicated hazards, indicated by the negative average values. Moreover, they perceived a high control about their health and low lack of control about diabetes and hypertension (Figure 1B), i.e., they believe that they can prevent the development of diabetes and hypertension.

When comparing the differences between sex, men presented a lower risk perception of gaining weight (−0.93) than women (−0.52; *p* = 0.016).

**Figure 2:**
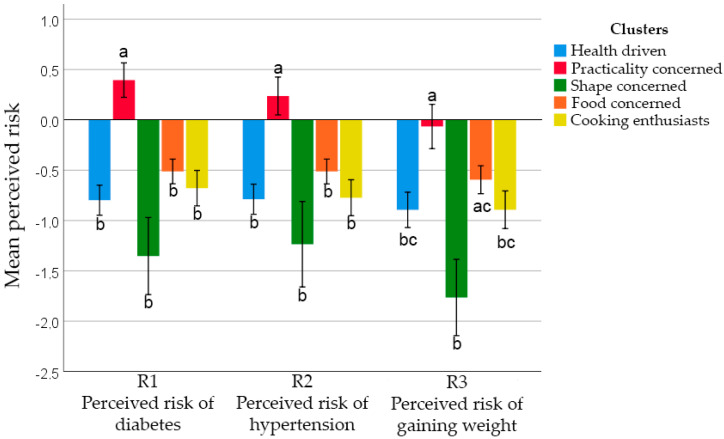
Perceived risk between FCQ clusters. Different letters within each risk perception indicate significant difference—Bonferroni’s test (*p* < 0.050).

However, individuals in cluster 2 (Practicality Concerned) indicated that they perceived a somewhat higher risk of having diabetes and hypertension and a regular risk for weight gain. Individuals in cluster 3 (Shape Concerned) seemed quite optimistic about the risk of weight gain, especially compared to clusters 2 and 4 (Figure 2).

Regarding the perception of health control (C1), in all clusters the mean value was higher than 4.1, with 1—Health Driven (5.0 ± 1.4); 2—Practicality Concerned (4.1 ± 1.6); 3—Shape Concerned (4.4 ± 2.3), 4—Food Concerned (5.1 ± 1.6); and 5—Cooking Enthusiasts (4.6 ± 1.7). Clusters 1 and 4 showed higher perceived control than clusters 2 and 5. There was no difference between the clusters regarding the control to prevent diabetes and hypertension (C2).

Table 5 shows the effects of the independent variables on the FCQ factors. Odds ratio (OR) < 1.0 denotes a negative effect (i.e., reduction) and OR > 1.0 denotes a positive effect (i.e., increasing) of the independent variables on the FCQ factors.

Socioeconomic factors affect, in different ways, the food choice motives. A negative effect of the male sex was observed in the scores of Nutritional Composition, Sensory Appeal, Health, Price, and Familiarity. However, with increasing age, men care more about Nutritional Composition. Having no children reduced individuals’ perceived importance of the Nutritional Composition, Convenience of Preparation, and Familiarity factors. According to the interactions observed in models 5 and 6, individuals without children tended to increase the importance of Familiarity and Convenience of Preparation with age. Notwithstanding, age did not affect these factors independently. The perceived control over health had a positive effect on the Nutritional Composition and Health factors.

A low education level and a high family income seemed to reduce the importance of food choice motives. There was a negative effect of less education on the scores of Sensory Appeal, Health, Familiarity, and Convenience of Purchase. In contrast, high income was related to lower scores for Price and Convenience of Preparation. However, an interaction between high income and age was also related to lower scores for Familiarity. It was decided not to keep the education and income variables in the same models in order to avoid collinearity, with only the one with higher explanatory power and better adjustment remaining.

No significant model emerged from the adjustment of the Mood factor.

## 4. Discussion

### 4.1. FCQ’s Structure

When CFA was applied in the present study, we changed the structure of the original questionnaire for a better model fit, with the exclusion of six items and rearrangement of the factors. These modifications are common and expected in different populations, and are explained by cultural differences, including regionalism, access to food, socioeconomic aspects, and food culture [23,34]. Even though the study conducted by Heitor et al. [27] was also carried out in Brazil, our results ended up differing in terms of the resulting FCQ structure. The differences in the regions where the studies were conducted, as well as the heterogeneity of the country, may explain these differences.

It appears to be a trend in some studies that the items related to Health, Natural Content, and Weight Control converge into a single factor [34,35]. In the present study, items of Natural Content and Weight Control were merged into the new Nutritional Composition factor, on the basis of the EFA. Although Health items were not included in this same factor, as shown in previous studies [34,35], it is worth mentioning that there was a moderate positive correlation between Health and Nutritional Composition. Moreover, it was found that Convenience can be differently perceived when food preparation or purchase is considered. The segregation of the Convenience factor was also observed in the Hungarian population [35]. It is suggested that, for some people, the complexity or time used to cook may not be a nuisance, but it may be necessary for those people that food is easily accessible for purchase.

### 4.2. Food Choice Motives

When comparing the eight FCQ factors, Sensory Appeal was the one with the highest average, indicating this factor as the leading food choice motive, which has been previously shown in other countries [23,35,36]. This factor is related to the pleasure of eating. Indeed, it is well established that food intake goes beyond the objective of nourishing the body [1], and hedonic issues related to food are also important in this process [37]. The central issue regarding the appreciation of sensory aspects is that food is considered a natural reward, since gratification from food consumption leads to the production of dopamine that activates reward and pleasure centers in the brain, especially considering highly palatable foods [15,38]. The problem lies when consumers overvalue sensory appeal and choose high palatable foods. This repetitive behavior, associated with an obesogenic environment, can override the signs of hunger and satiety, leading to excesses and less healthy food choices [15]. A positive hedonic interpretation of food increases the feeling of well-being and joy after eating and is associated with fullness during and after eating [39].

As examples of hyper-palatable foods, there are ultra-processed foods, which have a low cost and high convenience (of availability and preparation), making them an easy choice [40]. Examples of ultra-processed foods with these characteristics are soft drinks, chocolate, margarine, dehydrated soups, sausages, and instant noodles [41]. It is known that ultra-processed products dominate the food supply in high-income countries, and their consumption has overgrown in middle-income countries by replacing home-cooked meals with RTE foods [42]. There is some evidence that the high consumption of ultra-processed foods is harmful to health [43,44], due to high amounts of calories, fats, and sugars. Given this, the results of the present study reinforce the importance of attention to these aspects, since, as noted, health and an adequate nutritional composition are not the primary food choice motives. It is worth mentioning that the sensory aspects of eating includes a number of other aspects besides palatability, such as visual cues, smell, taste, and texture [45]. Therefore, health professionals and the food industry have a role on stimulating these other sensory aspects on less palatable and more healthy foods, making them as wanted as palatable foods, which would potentially contribute to better food choices and weight management.

### 4.3. Risk Perceptions and Food Choice Patterns

The individuals presented a low-risk perception of having diabetes, hypertension, and gaining weight, especially for the latter. In contrast, a high perceived control about their health was shown. Events perceived as controllable tend to be expressed with more optimism by people [46]. If the person thinks he/she is in control of the situation, he/she probably believes that his/her actions can increase the chances of a positive outcome [19]. This explains why the perception of risk about weight gain was lower than the risk perception of having diabetes and hypertension, despite Brazil ranking third in the world for the absolute number of obese adult men and the fifth position for the number of obese women during 2014 [47]. Some differences in the perceived risk were observed among different food choices clusters, which is further discussed.

As a result of the food choice motives, the studied population could be divided into five well-defined clusters, defining food choice patterns. The Food Concerned cluster was labeled as so, because in this cluster all the factors of the FCQ were somehow important for the choice of food and because it can be composed of people who pay attention to their food choices as a whole. In contrast, all other clusters had distinctions in the characteristics that define them.

The Cooking Enthusiasts cluster was characterized by individuals looking for accessible foods with excellent sensory characteristics, but they do not seem to care about the complexity of the preparation. The label assigned to this cluster was based on people who dedicate themselves to preparing meals and take pleasure in doing so. Home cooking is strongly encouraged by the Food Guide for the Brazilian Population of the Ministry of Health [41]. The pleasure in home cooking is an essential predictor of culinary skills, which correlate positively with the weekly consumption of vegetables and negatively with the consumption of “convenient foods” and RTE food [48]. It can be suggested that people in this cluster would potentially have positive health-related behavior.

The Practicality Concerned cluster is, in a way, antagonistic to the Cooking Enthusiasts cluster since the primary motive observed for food choices was the Preparation Convenience and the least important were related to Health and Nutritional Composition of the food. This importance attributed to Convenience may be associated with the choice of ultra-processed foods [49], whose consumption has been linked to poor quality of diet and, among other factors, to the increase in the prevalence of chronic diseases [50,51]. Individuals in the Practicality Concerned cluster have a lower perception of control over their health than individuals in the Health and Food Concerned clusters. Moreover, they have a higher perception of personal risk for diabetes and hypertension than other clusters and a neutral perception of the risk of weight gain. This may indicate that the members of this cluster may be aware of the possible harms of their food choices, and deliberately neglect this aspect. The low score of the Health factor in this cluster reinforces this hypothesis. Contrary to the Cooking Enthusiasts, the Practically Concerned cluster indicates a risky health-related behavior, and this is useful information considering the use of FCQ in research and clinical practice.

Individuals from the Shape Concerned cluster attributed importance to Nutritional Composition and Sensory Appeal but also attributed lower scores to the Health factor. This result provokes reflections about food composition being overrated, with particular attention to the amount of fat or caloric value, but not necessarily due to health concerns. Another hypothesis is that a food low on calories, fat, and without additives may be what this group of consumers understand as healthy food, especially due to the concern with weight or body shape for aesthetic reasons. This kind of thinking presents several implications in food behavior. For example, eating food perceived as healthy was previously associated with an increase in hunger [52], and a compensatory effect occurs when eating a perceived low-fat food in comparison to a perceived high-fat food [53], which can count for unsuccessful weight loss diets and be related to psychological distress. There are many diets extolled by the media, known as fad diets, in which the nutritional composition (carbohydrate, protein, or fat content), caloric value, and the final result of the diet on the body are often highlighted [54]. Moreover, information about diets is published in blogs and magazines, including menus, with the attraction of quick results, which can potentially put consumers who follow these recommendations without professional counseling at risk [55,56]. These considerations raise attention to the interdisciplinary aspect of food choices/behavior, which requires, consequently, interdisciplinary approaches in health and policy practices.

Although the score attributed to the Health factor by the individuals in the Shape Concerned cluster was lower, they were quite optimistic about their own risk of developing diabetes; hypertension; and above all, gaining weight. Individuals in this cluster believe that they may be healthy with the consumption of foods low in calories, fat, and additives. However, they fail to identify the importance of nutrients, fiber, and other motives such as mood and sensory appeal. When the stereotypes attributed to high- and low-fat consumption were investigated, a low-fat diet was associated with a “thin” and “healthy” person. In contrast, a high-fat diet was associated with “unhealthy” and “overweight” people, but who are happier [57]. These results demonstrate the dichotomy between health and eating pleasure, as well as the relationship between health and body weight with the nutritional composition of the food eaten. Probably, when concerned with aspects related to food, such as having little fat or low calories, individuals already believe that, even in an indirect way, they are taking a protective attitude towards their health, even if they do not think directly of health at the time of food choice.

### 4.4. Modelling Food Choice Motives

Finally, the use of linear models sought to evaluate the variables that affect the FCQ factors. It was observed that the importance of factors like Nutritional Composition and Health increased with age, as well as the interaction between male gender and age, increasing the importance given to Nutritional Composition. Data from 2008–2009 Household Budget Survey (*Pesquisa de Orçamentos Familiares*, in Portuguese) [10] showed that the consumption of ultra-processed foods reduces with increasing age. These changes in food consumption can be understood as a form of self-care. This self-care seems to increase over time when the feeling of being closer to the exposure of diseases linked to poor health habits is less latent. Younger people have a lower risk perception of adverse events that they understand as controllable, such as chronic noncommunicable diseases, and consequently, they exert riskier behaviors [58,59]. It was observed that the perception of control over health positively affected the factors of Health and Nutritional Composition, as well as scoring higher for the Health Driven and Food Concerned clusters. However, the relationship between practices and risk perception is complex. Practices understood as adequate might reduce the risk perception (self-efficacy). On the other hand, a low-risk perception might lead the individual to exert risky behaviors since the hazard is perceived as controllable (the illusion of control) [60].

The comparison between sexes indicated that higher scores were attributed by women to all the investigated food choice motives, as well as being male having an independent negative effect on the scores of Nutritional Composition, Sensory Appeal, Health, Price, and Familiarity. Higher scores were also attributed by women in London, with the attribution of greater importance to Natural Content and Weight Control being associated with a better quality of diet [49].

Men presented less risk perception to gain weight. Previous findings demonstrate that women tend to have higher body dissatisfaction and concern about weight gain [61,62]. Being female and being afraid of gaining weight are associated with the practice of diets, even in women with normal weight [61,63], which may be related to taking actions to avoid weight gain and thus reduce the perceived risk [64].

Women tend to have healthier food consumption than men, as well as more concern for food quality and healthcare, justifying this result [5,65,66]. These results may also be related to the fact that women are primarily responsible for preparing meals at home and have better culinary ability [48]. Women seem to be more health-conscious and have more pleasure in cooking than men [48]. According to the cited authors, men’s motivation to cook is possibly because they want to do it, instead of being an everyday obligation.

As expected, socioeconomic aspects such as income and education also affected food choice motives. According to the 2017–2018 POF [67], food was equivalent to 17.5% of the average consumption expenses of Brazilian families, with higher expenses for housing (36.6%) and transportation (18.1%). The survey cites a reduction in this percentage, highlighting that in the 2008–2009 survey, the contribution of food to consumption expenses was 19.8%. Therefore, although food still has a significant representation in Brazilian citizens’ expenses, this reduction may represent a greater interest in saving family resources, which may be related to the findings in the present study, since Price had the second-highest importance. This need to save money can also be reflected in the quality of the food, since most of the ultra-processed foods are also low cost [42].

## 5. Conclusions

It was possible to observe a heterogeneous profile of the motives for choosing food in Brazil. The choices were strongly linked to socioeconomic factors, but valuing sensorial aspects, with a well-presented role for tasty and nice-smelling food. For this reason, Sensory Appeal was the factor with the highest score. The factors of Price, Convenience of Purchase, and Health also showed higher averages than the other factors. The perception of control over health also affected the motives of food choice. Most participants perceived themselves as low risk for diabetes, hypertension, and weight gain, independently of their food choice motives. However, those more concerned with practicality when eating showed a less optimistic view about these hazards.

The identified clusters demonstrated that the factors must be understood together, facilitating the identification of patterns. Individuals with motivations geared to fitness (Shape Concerned) or quick meals (Practically Concerned) may have a poor diet, requiring more attention by health professionals. Moreover, traditional health messages and campaigns may not be effective for this audience.

The hierarchy of factors does seem to depend on the country in which the FCQ is applied. Factors such as education, income, age, and sex can affect the valuation of the factors. Therefore, this highlights the importance of the reproducibility of this type of research, investigating the reasons involved in food choices across different populations.

The interrelation of factors demonstrates the importance of public policies and health campaigns. Professionals in this sector need to have a comprehensive view of these aspects to have more effective practices. Policies for food access, information campaigns to raise awareness about nutritional quality, and factors related to the development of chronic diseases are essential, but equally as important to review how these messages are addressed. Thus, a focus on nutritional value and health alone may not be sufficient for everyone, with a potential strategy being to expand the approach to other factors.

## Figures and Tables

**Table 1:** Socioeconomic characteristics of the sample (*n* = 525).

Variable	Frequency (*n*)	Valid Percentage (%)
Age (years old)		
Young adults (18–34)	301	57.3
Adults (35–59)	167	31.8
Elderly (>60)	57	10.9
Marital status		
Single	298	56.8
Married	170	32.4
Divorced	33	6.3
Widower	24	4.6
Have children		
No	314	59.9
Yes	210	40.1
Education level		
Incomplete primary education	43	8.2
Complete primary education	30	5.7
Incomplete high school	21	4.0
Complete high school	67	12.8
Incomplete higher education	258	49.1
Complete higher education	106	20.2
Family income		
1 to 2 times the minimum wage *	99	19.1
Between 2 and 3 times the minimum wage	88	17.0
Between 3 and 4 times the minimum wage	70	13.5
Between 4 and 5 times the minimum wage	61	11.8
More than 5 times the minimum wage	199	38.5

For some variables, the total sum is less than 525 due to missing values. * Brazilian 2019 minimum wage per month = BRL 954.00 ≅ USD 261.36.

**Table 2:** Food Choice Questionnaire (FCQ)’s structure.

Factor	Items—“It Is Important to Me That the Food I Eat on a Typical Day...”	Loading	Mean (SD) *	Cronbach’s α
**Factor 1**	**Nutritional Composition**		**5.46 ^c^ (1.23)**	**0.863**
	Contains natural ingredients	0.724	5.80 (1.37)	
	Contains no additives	0.786	5.47 (1.60)	
	Contains no artificial ingredients	0.761	5.37 (1.65)	
	Is low in fat	0.612	5.51 (1.49)	
	Is low in calories	0.574	5.16 (1.58)	
	Helps me control my weight	0.544	5.57 (1.53)	
**Factor 2**	**Mood**		**5.48 ^c^ (1.15)**	**0.794**
	Helps me cope with stress	0.676	4.98 (1.78)	
	Helps me cope with life	0.737	5.22 (1.78)	
	Helps me relax	0.775	5.23 (1.58)	
	Makes me feel good	0.521	6.21 (1.14)	
	Cheers me up	0.710	5.76 (1.41)	
**Factor 3**	**Health**		**5.94 ^b^ (1.15)**	**0.833**
	Is nutritious	0.715	6.25 (1.19)	
	Is high in fibre and roughage	0.537	5.62 (1.47)	
	Contains a lot of vitamins and minerals	0.644	5.80 (1.34)	
	Is good for my skin/teeth/hair/nails etc.	0.420	5.66 (1.61)	
	Keeps me healthy	0.497	6.37 (1.00)	
**Factor 4**	**Sensory Appeal**		**6.19 ^a^ (0.90)**	**0.778**
	Looks nice	0.761	6.08 (1.27)	
	Has a pleasant texture	0.766	5.96 (1.32)	
	Tastes good	0.431	6.56 (0.82)	
	Smells nice	0.679	6.16 (1.20)	
**Factor 5**	**Price**		**6.01 ^b^ (1.02)**	**0.763**
	Is not expensive	0.767	5.86 (1.36)	
	Is cheap	0.803	5.79 (1.33)	
	Is good value for money	0.549	6.38 (0.98)	
**Factor 6**	**Preparation Convenience**		**5.59 ^c^ (1.31)**	**0.852**
	Is easy to prepare	0.727	5.77 (1.43)	
	Takes no time to prepare	0.899	5.47 (1.53)	
	Can be cooked very simply	0.866	5.52 (1.51)	
**Factor 7**	**Familiarity**		**4.99 ^d^ (1.34)**	**0.712**
	Is familiar to me	0.537	5.48 (1.51)	
	Is what I usually eat	0.710	5.45 (1.60)	
	Is like the food I ate when I was a child	0.696	4.03 (1.94)	
**Factor 8**	**Purchase Convenience**		**5.95 ^b^ (1.18)**	**0.625**
	Is easily available in shops and supermarkets	0.660	5.91 (1.47)	
	Can be bought in shops close to where I live or work	0.426	5.99 (1.30)	

* Scale from 1—totally disagree to 7—totally agree; different letters indicate significant differences in factors according to Bonferroni’s test (*p* < 0.050).

**Table 3:** Pearson’s correlation coefficient (*r*) between the FCQ factors (discriminant validity).

FCQ Factors		FCQ Factors						
	1	2	3	4	5	6	7	8
Nutritional composition (1)	1.000						.	
Mood (2)	0.398	1.000						
Health (3)	0.684	0.477	1.000					
Sensory Appeal (4)	0.487	0.353	0.400	1.000				
Price (5)	0.384	0.291	0.271	0.375	1.000			
Convenience of Preparation (6)	0.192	0.258	0.226	0.191	0.422	1.000		
Familiarity (7)	0.425	0.367	0.334	0.496	0.317	0.293	1.000	
Convenience of Purchase (8)	0.252	0.307	0.329	0.336	0.475	0.415	0.284	1.000

All correlations presented *p* < 0.001.

**Table 4:** Average (SD) FCQ factor scores for each cluster.

FCQ Factors	Clusters					*F*-Value *
	1—Health Driven (*n* = 106)	2—Practicality Concerned (*n* = 77)	3—Shape Concerned (*n* = 18)	4—Food Concerned (*n* = 209)	5—Cooking Enthusiasts (*n* = 88)	
Sensory Appeal	5.22 ^c,B^ (0.94)	6.04 ^b,A^ (0.84)	6.33 ^ab,A^ (1.10)	6.69 ^a,A^ (0.42)	6.24 ^b,A^ (0.73)	72.70
Price	5.15 ^c,BC^ (1.03)	5.91 ^b,A^ (0.90)	6.01 ^b,AB^ (1.33)	6.62 ^a,A^ (0.52)	5.67 ^b,B^ (0.98)	49.45
Purchase Convenience	5.18 ^c,B^ (1.15)	5.96 ^b,A^ (0.92)	3.38 ^d,C^ (1.74)	6.58 ^a,A^ (0.63)	5.84 ^b,B^ (0.99)	89.24
Health	5.86 ^b,A^ (0.87)	4.62 ^c,C^ (1.06)	4.76 ^c,BC^ (1.26)	6.58 ^a,A^ (0.48)	5.84 ^b,B^ (0.77)	50.93
Preparation Convenience	5.51 ^b,AB^ (1.00)	6.07 ^a,A^ (0.90)	4.42 ^c,BC^ (1.62)	6.31 ^a,B^ (0.65)	3.78 ^c,D^ (1.06)	27.77
Mood	4.74 ^c,C^ (1.13)	5.18 ^b,B^ (1.25)	4.94 ^bc,BC^ (1.25)	6.10 ^a,B^ (0.82)	5.17^b,C^ (0.92)	102.01
Nutritional Composition	5.13 ^b,BC^ (0.99)	3.92 ^d,D^ (0.91)	5.80 ^a,AB^ (1.22)	6.37 ^a,B^ (0.64)	5.13 ^c,C^ (1.00)	79.57
Familiarity	4.14 ^b,D^ (1.21)	4.62 ^b,C^ (1.11)	4.90 ^b,BC^ (1.43)	5.77 ^a,C^ (1.06)	4.29 ^b,D^ (1.26)	38.00
*F*-value **	115.21	19.78	32.07	118.10	42.15	-

* One-way ANOVA test among clusters with *p* < 0.001; N total = 498. a, b, c, d—homogeneous groups among clusters according to Bonferroni’s test (*p* < 0.050), to be read horizontally for each factor. ** Repeated measures ANOVA among the factors with *p* < 0.001. A, B, C, D—homogeneous groups among factors according to *Bonferroni’s* test (*p* < 0.050), to be read vertically for each cluster.

**Table 5:** Odds ratio values of the mixed linear models developed for the final FCQ constructs.

Independent Variables	Dependent Variables						
	Model 1—Nut Comp	Model 2—Sens App	Model 3—Health	Model 4—Price	Model 5—Fam	Model 6—Prep Conv	Model 7—Purch Conv
	Odds Ratio [CI 95%]						
Sex (base value = female)	0.32 (0.21–0.49)	0.72 (0.62–0.84)	0.66 (0.55–0.78)	0.83 (0.69–0.99)	0.72 (0.59–0.88)	-	-
Age (years)	1.01 (1.00–1.02)	1.01 (1.00–1.02)	1.01 (1.00–1.02)	-	1.00 * (0.99—1.01)	-	-
Children in household (base level = no)	0.72 (0.54–0.94)	-	-	-	0.31 (0.17–0.58)	0.37 (0.20–0.67)	-
Income (base level = low income)	0.88 * (0.73–1.06)	-	-	0.62 (0.52–0.75)	-	0.81 (0.68–0.97)	-
Perceived health control (scale from 1 to 7)	1.10 (1.04–1.16)	-	1.11 (1.06–1.17)	-	-	-	-
Education level (base level = high education level)	NS	0.77 (0.63–0.93)	0.80 (0.64–1.00)	-	0.53 (0.41–0.69)	-	0.54 (0.45–0.66)
Interaction (male sex and age)	1.02 (1.01–1.03)	-	-	-	-	-	-
Interaction (high income and age)	-	-	-	-	0.99 (0.99–1.00)	-	-
Interaction (have no children and age)	-	-	-	-	1.02 (1.01–1.04)	1.01 (1.00–1.02)	-

OR = odds ratio; CI 95% = confidence interval of 95%. Bold values indicate significant OR. * Adjust variable. Nut Comp = Nutritional Composition; Sens App = Sensory Appeal; Fam = Familiarity; Prep Conv = Preparation Convenience; Purch Conv = Purchase Convenience.
